# Comparative Efficacy of the Novel Diarylquinoline TBAJ-876 and Bedaquiline against a Resistant *Rv0678* Mutant in a Mouse Model of Tuberculosis

**DOI:** 10.1128/AAC.01412-21

**Published:** 2021-11-17

**Authors:** Deepak Almeida, Paul J. Converse, Si-Yang Li, Anna M. Upton, Nader Fotouhi, Eric L. Nuermberger

**Affiliations:** a Center for Tuberculosis Research, Department of Medicine, Johns Hopkins University grid.21107.35grid.471401.7 School of Medicine, Baltimore, Maryland, USA; b Global Alliance for TB Drug Development, New York, New York, USA; c Department of International Health, Johns Hopkins Bloomberg School of Public Health, Baltimore, Maryland, USA

**Keywords:** *Mycobacterium tuberculosis*, TBAJ-876, bedaquiline, linezolid, mouse, pretomanid, resistance, tuberculosis

## Abstract

Bedaquiline (BDQ, B) is the first-in-class diarylquinoline to be approved for treatment of tuberculosis (TB). Recent guidelines recommend its use in treatment of multidrug- and extensively drug-resistant tuberculosis (MDR/XDR-TB). The newly approved regimen combining BDQ with pretomanid and linezolid is the first 6-month oral regimen proven to be effective against MDR/XDR-TB. However, the emergence of BDQ resistance, primarily due to inactivating mutations in the *Rv0678* gene encoding a repressor of the MmpS5-MmpL5 transporter, threatens to undermine the efficacy of new BDQ-containing regimens. Since the shift in MIC due to these mutations is relatively small (2–8×), safer, and more potent, diarylquinoline analogues may be more effective than BDQ. TBAJ-876, which is in phase 1 trials, has more potent *in vitro* activity and a superior pre-clinical safety profile than BDQ. Using a murine model of TB, we evaluated the dose-dependent activity of TBAJ-876 compared to BDQ against the wild-type H37Rv strain and an isogenic *Rv0678* loss-of-function mutant. Although the mutation affected the MIC of both drugs, the MIC of TBAJ-876 against the mutant was 10-fold lower than that of BDQ. TBAJ-876 at doses ≥6.25 mg/kg had greater efficacy against both strains compared to BDQ at 25 mg/kg, when administered alone or in combination with pretomanid and linezolid. Likewise, no selective amplification of BDQ-resistant bacteria was observed at TBAJ-876 doses ≥6.25 mg/kg. These results indicate that replacing BDQ with TBAJ-876 may shorten the duration of TB treatment and be more effective in treating and preventing infections caused by *Rv0678* mutants.

## INTRODUCTION

Bedaquiline (BDQ) is the first-in-class diarylquinoline that, in 2012, became the first drug from a novel class to be approved by the U.S. Food and Drug Administration (FDA) for treatment of tuberculosis (TB) in four decades (1). It acts by selectively inhibiting mycobacterial ATP synthase and exerts strong bactericidal and sterilizing activity against Mycobacterium tuberculosis (2–13). Recent World Health Organization guidelines (14) recommend the use of BDQ in regimens to treat multidrug resistant (MDR) and extensively drug-resistant (XDR) TB. Moreover, novel regimens combining BDQ with another newly FDA-approved drug pretomanid (PMD) (15) have shown potential to reduce the duration of treatment in murine models of TB (16, 17) and in humans (18, 19). In the recent Nix-TB trial, the combination of BDQ, PMD, and linezolid (LZD) (regimen abbreviated as BPaL) resulted in successful outcomes in 90% of XDR-TB and difficult-to-treat MDR-TB patients with just 6 months of treatment (18). In the NC-005 trial, faster sputum culture conversion was observed among MDR-TB patients receiving the combination of BDQ, PMD, moxifloxacin (MXF), and pyrazinamide (PZA) (regimen abbreviated as BPaMZ) compared to those treated with the first-line standard-of-care regimen (SOC) in drug-susceptible (DS) TB patients (19), prompting the ongoing SimpliciTB trial (ClinicalTrials.gov identifier: NCT03338621) to determine if a 4-month duration of BPaMZ is non-inferior to the 6-month SOC regimen in DS-T.

Despite the effectiveness of BDQ against TB and success of BDQ-containing regimens thus far, BDQ has limitations. Its highly lipophilic nature (20) contributes to its long terminal half-life of 5–6 months, very high protein binding (>99.9%), and relatively slow partitioning into caseous lesions (21). It also inhibits cardiac hERG/IKr potassium channels, resulting in potential for QTc prolongation that can be additive with other drugs (21). The emergence of BDQ resistance has also threatened to undermine the efficacy of BDQ-containing regimens. Initial efforts to select drug–resistant mutants *in vitro* identified mutations in the *atpE* gene as the main cause of resistance (13). However, mutations in the *Rv0678* gene are far more common causes of resistance in murine TB models and in the clinical setting (22–26). Rv0678 is a transcriptional repressor of the MmpS5-MmpL5 efflux system, and inactivating mutations in *Rv0678* de-repress *mmpS5*-*mmpL5* expression, resulting in increased efflux of BDQ from the cell (27). Although the resultant increase in MIC is relatively small, *Rv0678* mutants are readily selected *in vivo* by both BDQ and clofazimine (CFZ) (22, 27). Of great concern is the fact that *Rv0678* mutants with reduced BDQ susceptibility have been isolated from MDR/XDR-TB patients without known prior exposure to BDQ or CFZ (22, 28, 29), raising the possibility that additional factors may contribute to their selective amplification. The emergence of *Rv0678* mutants could undermine the promising clinical efficacy of BDQ-containing regimens. Increasing the BDQ dose could help to overcome some of the resistance conferred by *Rv0678* mutations. However, potential safety concerns, including QTc prolongation, limit enthusiasm for testing higher BDQ doses (30).

Efforts to develop diarylquinoline analogues with superior potency, lower lipophilicity, and superior cardiovascular risk profiles have led to clinical development of two promising candidates, TBAJ-587 and TBAJ-876 (31–35). Both drugs are now in phase 1 trials (ClinicalTrials.gov identifier: NCT04493671). Although mutation of *Rv0678* reduces the *in vitro* activity of TBAJ-876 and TBAJ-587 to a similar degree as BDQ, these analogues remain more potent than BDQ against such mutants and therefore may be more effective at killing them or preventing their selection during treatment. We previously compared the potency of TBAJ-587 and BDQ (26) alone and in combination regimens in a mouse model of TB and found TBAJ-587 to be significantly more bactericidal when tested at the same dose as BDQ and a promising candidate for BDQ replacement.

In the current experiment, we compared the dose-dependent effects of BDQ and TBAJ-876 against an *Rv0678* loss-of-function mutant and the wild-type H37Rv parental strain, to determine the impact of inactivating *Rv0678* mutations on the activity of TBAJ-876 and its contribution to regimen efficacy when replacing BDQ in the BPaL regimen. The results indicate that replacing BDQ with TBAJ-876 could increase regimen efficacy against wild-type M. tuberculosis as well as *Rv0678* mutants and potentially reduce the emergence of resistance to diarylquinolines and companion drugs.

## RESULTS

### Bacterial strains and mouse infection model.

The experimental scheme indicating the regimens used against the wild-type strain, M. tuberculosis H37Rv, and an isogenic mutant with an IS6110 insertion in the *Rv0678* gene is shown in Table S1. Using the broth macrodilution method in 7H9 media, the MICs of BDQ and TBAJ-876 against the H37Rv strain were 0.03 and 0.006 μg/ml, respectively, while against the *Rv0678* mutant, the MICs were 0.25 μg/ml and 0.025 μg/ml, respectively. The MIC of PMD was 0.25 μg/ml against both strains. The MIC for LZD was not performed against the *Rv0678* mutant used to infect mice. However, a previous whole genome analysis did not reveal mutations associated with resistance to LZD or PMD (36).

One day after high-dose aerosol infection of BALB/c mice with either strain, mean (±SD) lung CFU (CFU) counts were 4.07 ± 0.07 log_10_ for H37Rv and 4.05 ± 0.01 for the *Rv0678* mutant (Tables S2 and S3). Mean CFU counts increased over the following 2 weeks to 7.61 ± 0.22 and 7.17 ± 0.09, respectively, when treatment began. Untreated mice were euthanized after developing clinical signs of progressive infection 3 weeks into the treatment period, in accordance with the approved animal care and use protocol. The mean lung CFU counts had increased to 9.22 ± 0.28 and 8.84 ± 0.15 in lungs of those mice infected with H37Rv and the *Rv0678* mutant, respectively.

### Response to treatment. (i) Mice infected with wild-type M. tuberculosis H37Rv.

**FIG 1:**
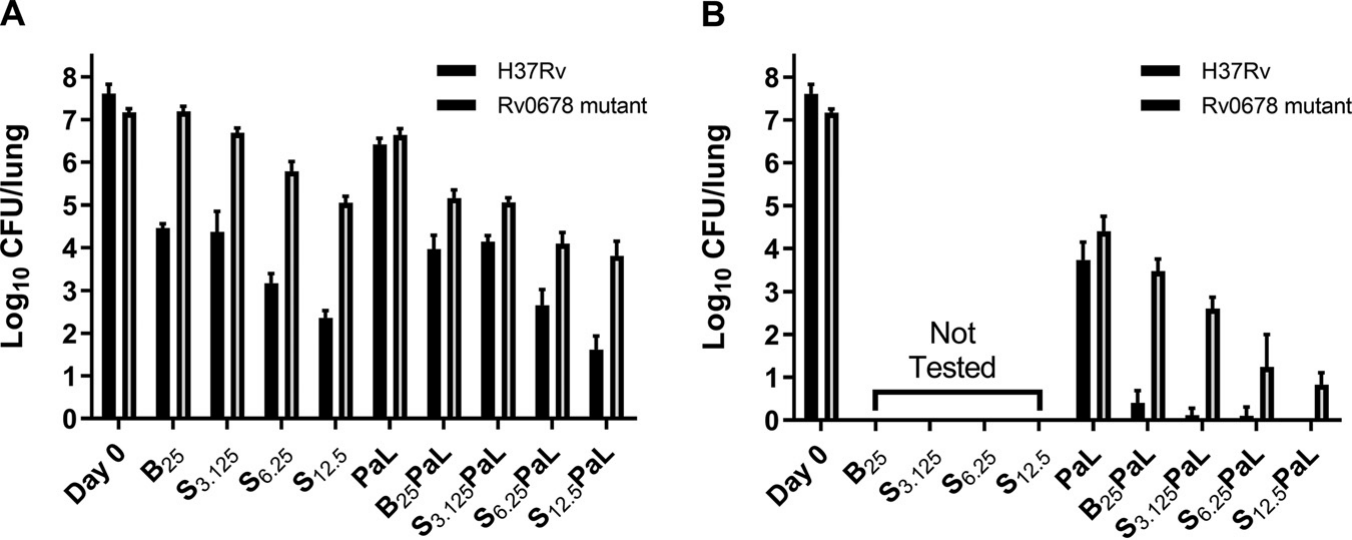
TBAJ-876 (S) shows dose-dependent activity and is more active than bedaquiline (B) at 6.25 and 12.5 mg/kg doses, either alone or in combination with pretomanid and linezolid (PaL) against wild-type M. tuberculosis (black bars) and against M. tuberculosis with an *Rv0678* mutation (gray bars). (A) Activity of monotherapy and combination regimens following 1 month of treatment. (B) Activity combination regimens following 2 months of treatment.

As shown in Fig. 1 and Table S2, after 1 month of treatment, BDQ at 25 mg/kg alone reduced the mean lung burden by 3.15 log_10_ compared to Day 0 (D0) and was more active than the combination of PMD and LZD (PaL). When added to PMD and LZD, BDQ reduced the mean CFU count by an additional 2.5 log_10_ (i.e., BPaL versus PaL). Treatment with TBAJ-876 (S) showed dose-dependent activity, with the lowest dose of 3.125 mg/kg showing similar activity to BDQ at 25 mg/kg (*P* > 0.999), while increasing the dose to 6.25 and 12.5 mg/kg significantly increased the activity over BDQ (*P* < 0.0001). A similar observation was made when TBAJ-876 was combined with PaL, with S_3.125_PaL showing a similar reduction in CFU as B_25_PaL (*P* > 0.999), while increasing the dose of TBAJ-876 to 6.25 and 12.5 mg/kg significantly increased activity over the 3.125 mg/kg dose (*P* < 0.0001). Increasing the treatment duration to 2 months in the combination groups further reduced CFU and showed continued contribution to killing from BDQ. The mean CFU count in the B_25_PaL group was 0.41 ± 0.28 log_10_ CFU/lung with one mouse having no detectable CFU (limit of detection < 2 CFU). When S_3.125_ or S_6.25_ replaced B_25_, the mean CFU counts were 0.12 ± 0.16 and 0.10 ± 0.21 log_10_, with only 2 and 1 of 5 mice having detectable CFU (limit of detection 1 CFU), respectively. S_12.5_PaL rendered all mice culture-negative at M2.

### (ii) Mice infected with M. tuberculosis H37Rv with an *Rv0678* mutation.

As shown in Fig. 1 and Table S3, dose-dependent reductions in lung CFU were observed with TBAJ-876 monotherapy against the *Rv0678* mutant as well. However, both BDQ and TBAJ-876 were significantly less active against the mutant than against the wild-type H37Rv strain. BDQ at 25 mg/kg did not reduce the mean CFU count at all compared to D0, but it did prevent the death of mice when compared to the untreated group. All three doses of TBAJ-876 were significantly more active than BDQ at 25 mg/kg (*P* = 0.0097 for S_3.125_; *P* < 0.001 for S_6.25_ and S_12.5_). PaL had a somewhat smaller effect size (i.e., smaller log kill compared to D0) against the *Rv0678* mutant than against H37Rv. Both BDQ and TBAJ-876 significantly (*P* < 0.0001) increased the activity of PaL at M1. As expected, both BDQ and TBAJ-876 contributed a smaller effect in combination with PaL against the *Rv0678* mutant than against the H37Rv strain. At M2, B_25_PaL was significantly better than PaL (*P* = 0.0287), as was TBAJ-876 at any dose (*p* <0.0001). All regimens combining TBAJ-876 with PaL were significantly better than B_25_PaL (*P* = 0.0450 for S_3.125_PaL; *P* < 0.0001 for S_6.25_PaL and S_12.5_PaL).

### Selection of drug-resistant mutants. (i) Mice infected with wild-type M. tuberculosis H37Rv.

At D0, the mean frequency of CFU able to grow on agar containing 0.125 μg/ml of BDQ was 4.6 × 10^−6^ or approximately 0.00046% of the total population. Plates containing BDQ 0.06 μg/ml were used to quantify the resistant subpopulation at subsequent time points based on prior evidence of its effectiveness for quantifying mutants with low-level resistance to BDQ in treated mouse lungs (37) in which BDQ carried over in lung homogenates adds to the total concentration of BDQ in the plates (26). Among mice infected with M. tuberculosis H37Rv and treated with BDQ monotherapy at 25 mg/kg for 1 month, one of five mice showed selection of BDQ-resistant CFU (1.29% of total CFU) (Table S4). In the TBAJ-876 monotherapy groups, for all three doses tested, none of the mice showed growth of CFU on BDQ-containing agar, indicating that all three doses prevented the selection of spontaneous BDQ-resistant mutants. Among the combination groups, none of the mice receiving BDQ or TBAJ-876 in combination with PaL showed the presence of BDQ-resistant CFU at 0.06 μg/ml at either M1 or M2. However, there was an indication of selective amplification of CFU able to grow on BDQ at 0.06 μg/ml in the PaL-treated group even though these mice were not exposed to BDQ. At M1, 4 of 5 mice receiving PaL harbored CFU on BDQ-containing plates, representing from 0.0066% to 0.009% (mean 0.008%) of total CFU, or a 10-to-20-fold increase from the baseline value of 0.00046%. At M2, 3 of 5 mice harbored CFU able to grow on BDQ-containing plates, representing 0.18%, 0.43%, and 1% of the total CFU. There was no evidence of selective amplification in the remaining 2 mice. Two or three colonies were isolated from the BDQ-containing plate for each mouse, and MICs of BDQ, PMD, and LZD were determined, with the H37Rv parent as control. All three colonies from each of two mice were resistant to PMD (MICs ≥2 μg/ml, versus 0.25 μg/ml for H37Rv) but not BDQ, while both colonies from the third remaining mouse were resistant to BDQ (MICs 0.5 μg/ml, versus 0.0625 μg/ml for H37Rv) but not PMD. None of the isolates were resistant to LZD (MICs 0.5 μg/ml). Whole genome sequencing revealed a mutation in *fgd* (*Rv0407*; Gly304Val) in one PMD-resistant isolate and a mutation in *fbiD* (*Rv2983*; Leu60Pro) in 1 of 2 colonies from the other PMD-resistant isolate. Mutations in each of these genes have been previously documented to be associated with PMD resistance (38–40). Both colonies from the BDQ-resistant isolate harbored an *Rv0678* mutation (Gly66Arg).

To further test whether BDQ-resistant mutants might have reduced susceptibility to PMD, MICs were determined against the H37Rv strain and additional *Rv0678* and *pepQ* mutants with reduced BDQ susceptibility that were previously selected in the H37Rv background, including two isolates with *Rv0678* mutations (+A in Tyr99 and +G in Gly65) previously associated with BDQ resistance in clinical isolates (41) and three isolates with *pepQ* mutations (+G in Arg271, Leu23Pro, and -tgaccgtgg in Val322). BDQ MICs were 1 μg/ml against each mutant, compared to 0.06 μg/ml against the H37Rv parent strain. PMD MICs were 0.25 μg/ml for all mutants, and the H37Rv parent strain indicating that the BDQ-resistant mutants had similar susceptibility to PMD as the H37Rv parent.

At D0, the mean frequency of CFU able to grow on agar containing 2 μg/ml of PMD was 4.3 × 10^−5^ or approximately 0.0043% of the total population. Quantification of the PMD-resistant subpopulation in H37Rv-infected mice was performed only at baseline to compare with the baseline resistance in the *Rv0678* mutant-infected group. Subsequent assessment of PMD resistance in H37Rv-infected mice at M1 and M2 was not planned because it may be confounded by BDQ carryover.

### (ii) Mice infected with M. tuberculosis H37Rv with an *Rv0678* mutation.

The baseline frequency of spontaneous resistance to BDQ at 1 μg/ml was 1 × 10^−5^ or approximately 0.0012% at the start of treatment (D0). Selective amplification of CFU able to grow on this higher concentration of BDQ did not occur in any group (Table S5). The baseline frequency of resistance to PMD at 2 μg/ml among mice infected with the *Rv0678* mutant at D0 was 8 × 10^−5^, or approximately 0.0077% of the total population, similar to what was seen among mice infected with H37Rv. At M1, PMD-resistant CFU were isolated from 4 of 5 mice treated with BDQ 25 mg/kg, 1 of 5 mice treated with TBAJ-876 3.125 mg/kg, and all 5 mice treated with PaL. However, the proportions of resistant CFU within the total population were similar to baseline levels, indicating no enrichment. In the B_25_PaL-treated group, 1 of 4 assessable mice harbored PMD-resistant CFU at a proportion that was between 0.01% and 0.5%, but could not be determined more precisely due to apparent BDQ carryover. In the S_3.125_PaL-treated group, 1 of 5 mice harbored PMD-resistant CFU above the baseline value at 0.17%, indicating a 20-fold increase. At M2, PMD-resistant CFU were isolated only from the PaL and S_3.125_PaL groups. In the PaL-treated group, 4 of 5 mice showed resistant CFU with an average frequency of 0.036%, a 4-to-5-fold increase from baseline. In the S_3.12_PaL-treated mice, 2 of 5 mice harbored PMD-resistant CFU at elevated proportions of 2.22% and 3.03%, indicating the lowest dose of TBAJ-876 allowed selective amplification of PMD-resistant mutants in an *Rv0678* mutant background when combined with PaL. However, it should be noted that the absolute PMD-resistant CFU count was very low in these mice (i.e., 5–10 CFU per lung) at M2. Similar numbers of PMD-resistant CFU in the BPaL arm could have been obscured by BDQ carryover.

## DISCUSSION

The treatment of MDR/XDR-TB is now greatly improved by introduction of BDQ and the fully oral short-course BPaL regimen (14, 18). However, the increasing frequency of baseline and acquired BDQ resistance mediated by *Rv0678* mutations may undermine the efficacy of BDQ and is a cause for great concern (18, 22, 24, 25, 28, 29, 42). Use of more potent next-generation diarylquinolines with improved pharmacological and safety profiles may better treat and prevent infections with *Rv0678* mutants. TBAJ-587 and TBAJ-876 are 3,5-dialkoxypyridine analogues of bedaquiline with such superior profiles (34) and are now in phase I clinical trials (35). TBAJ-876 retains bedaquiline's activity against subunits c and ε of the F-ATP synthase (43). In a previous study we reported the superior efficacy of TBAJ-587 compared to BDQ alone and in the BPaL and BPaMZ regimens against both the wild-type H37Rv strain and an isogenic *Rv0678* mutant (26). In the current experiment, we studied the effects of replacing BDQ with TBAJ-876, which has more favorable PK and toxicity profile than BDQ (31, 35). The lowest dose of TBAJ-876 (3.125 mg/kg) reduced lung CFU counts to a similar degree as BDQ 25 mg/kg. Furthermore, TBAJ-876 doses of 6.25 and 12.5 mg/kg were significantly better than BDQ at reducing the CFU burden, suggesting that it may have the potential to shorten the duration needed to prevent relapse at these doses when substituted for BDQ. Sutherland et al. (34) previously evaluated plasma exposures for these drugs in BALB/c mice after a single oral dose of 20 mg/kg and reported AUC_0-inf_ values of 20.9 mg-h/liter for BDQ and 5.61 mg-h/liter for TBAJ-876, compared to their MICs of 0.04 and 0.004 μg/ml, respectively. Thus, the greater efficacy of TBAJ-876 at 6.25 mg/kg compared to BDQ at 25 mg/kg is attributable to its superior potency against M. tuberculosis and despite achieving lower drug exposures. Sutherland et al. (34) also reported an AUC_0-inf_ value of 1.72 mg-h/liter for TBAJ-587 after a 20 mg/kg dose and an MIC of 0.006 μg/ml. These results suggest that TBAJ-876 at 6.25 mg/kg and TBAJ-587 at 25 mg/kg would produce similar AUC/MIC values and therefore that TBAJ-876 would be roughly 4-fold more potent *in vivo*. Comparing log kill results at M1 in our prior study with TBAJ-587 (26) with those in the current study with TBAJ-876, we indeed observed that TBAJ-587 at 25 mg/kg had efficacy similar to TBAJ-876 at 6.25 mg/kg.

Similar to observations with TBAJ-587 (26), the efficacy of both BDQ and TBAJ-876 was reduced in mice infected with the *Rv067*8 mutant. However, unlike against H37Rv infection where TBAJ-876 3.125 mg/kg had similar efficacy to BDQ, all doses of TBAJ-876 were significantly better than BDQ at reducing the CFU burden of the *Rv0678* mutant, suggesting that the *Rv0678* mutation does not affect TBAJ-876 to the same degree as it does BDQ. This conclusion is also supported by the somewhat smaller shift in TBAJ-876 MIC against the mutant compared to the shift in BDQ MIC. At 25 mg/kg/day, BDQ lost its bactericidal activity against the *Rv0678* mutant, whereas TBAJ-876 exhibited clear-cut bactericidal activity at doses ≥6.25 mg/kg as monotherapy and a modest, but still significant, 0.5 log_10_ CFU reduction at 3.125 mg/kg. As combination therapy, S_6.25_PaL and S_12.5_PaL were practically as effective against the mutant as BPaL was against the wild-type H37Rv strain.

In mice infected with H37Rv and treated with BDQ 25 alone, resistance to BDQ emerged in 1 of 5 mice after 1 month of treatment, but no resistance was seen in mice treated with any dose of TBAJ-876 alone after 1 month, indicating that it may be more effective at preventing selection of BDQ-resistant mutants. Selection of BDQ-resistant CFU occurred in all BDQ-treated mice infected with H37Rv in our recent study (26). The lower proportion of mice with resistance observed in the current study is likely attributable to the approximately 10-fold lower frequency of spontaneous BDQ-resistant mutants present at baseline compared to the prior study. As reported previously with TBAJ-587 (26), we did not observe selection of higher-level, or “second-step,” BDQ resistance despite treating the *Rv0678* mutant infection with BDQ or TBAJ-876 alone. Inability to readily select more resistant *atpE* mutants may be due to a low frequency of viable spontaneous *atpE* mutants and to the fitness costs observed *in vivo* (37). Although *atpE* mutations have been identified in a small number of BDQ-resistant clinical isolates to date (44, 45), *Rv0678* mutations have been much more prevalent. Therefore, overcoming *Rv0678*-mediated resistance with more potent diarylquinolines like TBAJ-876 could greatly extend the utility and longevity of this important new class of drugs.

In our recent study with TBAJ-587 (26), selective amplification of PMD-resistant CFU (to 0.6% of total CFU, on average) occurred at M2 in all 5 mice infected with the *Rv0678* mutant and treated with BPaL, suggesting that inadvertent treatment of patients infected with an *Rv0678* mutant with BPaL could lead to a dangerous new form of multidrug resistance defined by resistance to diarylquinolines and nitroimidazoles, including delamanid (26, 38, 46). In the present study, the PMD-resistant population was enriched compared to baseline in 3 of 10 mice infected with the *Rv0678* mutant and treated with S_3.125_PaL at M1 and M2, reaching to 0.5%, on average, of the total CFU at M2. A similar degree of resistance may have occurred in at least one mouse treated with BPaL. BDQ carryover, which was evident at M1, may have obscured evidence of such selective amplification in other mice. Higher doses of TBAJ-876 were not associated with isolation of any PMD-resistant CFU. Taking both studies together, the results support replacing BDQ with TBAJ-876 or TBAJ-587 doses that yield more efficacious exposures than BDQ to limit emergence of both diarylquinoline and PMD resistance.

Finally, in mice infected with wild-type H37Rv and treated with PaL, we observed enrichment of the bacterial population able to grow on BDQ 0.06 μg/ml. At M2, 3 of 5 mice harbored isolates in which 0.18–1% of the total CFU grew on BDQ-containing plates. However, subsequent MIC testing revealed that only 1 of these 3 isolates was BDQ-resistant. The implication of this result is not clear, since neither *Rv0678* nor *pepQ* mutants are known to have reduced susceptibility to PMD, and treatment with PaL in the *Rv0678* mutant background did not show evidence of selection of more resistant *atpE* mutants. We did not observe enrichment of CFU growing on BDQ-containing plates in our previous study (26), although PaL treatment of H37Rv infection was not extended beyond M1. However, in that study as well as the present study, PaL treatment resulted in somewhat smaller effects against *Rv0678* mutant infections compared to H37Rv infections, despite there being no apparent differences in PMD or LZD MICs against *Rv0678* mutants compared to the parental H37Rv strain. We cannot exclude the possibility that PaL treatment is somewhat less effective against *Rv0678* mutants and has potential to select for these or other low-level BDQ-resistant or BDQ-tolerant CFU *in vivo*. Further study of this phenomenon may be warranted.

In conclusion, TBAJ-876 has dose-ranging bactericidal activity against wild-type H37Rv at much lower doses compared to BDQ and was more active against the isogenic BDQ-resistant *Rv0678* mutant as well. TBAJ-876 at 6.25 and 12.5 mg/kg in combination with PaL was found to be as effective against the *Rv0678* mutant as BPaL was against the wild-type H37Rv strain. Therefore, if exposures similar to the 6.25 mg/kg dose in mice can be achieved in patients, TBAJ-876 may provide a more effective diarylquinoline alternative to BDQ for treatment of TB, including prevention and treatment of infections caused by *Rv0678* mutants.

## MATERIALS AND METHODS

### Bacterial strains.

M. tuberculosis H37Rv, and a spontaneous BDQ-resistant mutant with an IS*6110* insertion in *Rv0678* at aa16/nt49, were used to infect mice in this study. The *Rv0678* mutant was isolated along with *pepQ* mutants in a prior study (36), but the responsible mutation was not identified until later. The *Rv0678* mutation and the absence of other mutations in genes associated with drug resistance were confirmed by whole genome sequencing.

### MIC testing.

The broth macrodilution method in complete 7H9 media without Tween 80 using polystyrene tubes and drug concentrations in doubling dilutions was used to determine the MICs against each of the strains used to infect mice, isolates growing on drug-containing plates after treatment, and mutants in our collection that were previously selected in the H37Rv background. The concentration ranges used were as follows: BDQ 0.0017 – 1.0 μg/ml; TBAJ-876 0.00075 – 0.1 μg/ml; PMD 0.03 – 2.0 μg/ml; LZD 0.125 – 8 μg/ml. We also determined the MICs of PMD and BDQ against H37Rv, *Rv0678* isolates having insertions at Tyr99 (+A) and Gly65 (+G), and *pepQ* mutants (+G at Arg271, Leu23Pro and -tgaccgtgg at Val322) previously selected in the H37Rv background in mice (unpublished). The concentration range used for PMD was 0.007–2 μg/ml and for BDQ 0.007–4 μg/ml.

### Infection model.

All animal procedures were conducted according to relevant national and international guidelines and approved by the Johns Hopkins University Animal Care and Use Committee. Female BALB/c mice, 6 wks old, were aerosol-infected with approximately 4 log_10_ CFU of each M. tuberculosis strain in two separate runs using a log phase culture with OD_600_ of approximately 0.8 (D-14). Treatment started 2 weeks later (D0). Mice were sacrificed for lung CFU counts on D-13 and D0 to determine the number of CFU implanted and the number present at the start of treatment, respectively.

### Antibiotic treatment.

Mice were randomized to one of 10 treatment groups (Table S1): untreated, B_25_, S_3.125_, S_6.25_ and S_12.5_, Pa_30_L_50_, B_25_PaL, S_3.125_PaL, S_6.25_PaL and S_12.5_PaL. The drug doses are indicated in subscripts after the drug abbreviations. BDQ and TBAJ-876 were formulated in 20% hydroxypropyl-β-cyclodextrin solution acidified with 1.5% 1N HCl. Pa was prepared in the CM-2 formulation as previously described (47). L was prepared in 0.5% methylcellulose. Drugs were administered by gavage, 5 days per week. BDQ or TBAJ-876 was given once daily. In all combinations, Pa 30 mg/kg (60 mg/kg total daily dose) and L 50 mg/kg (100 mg/kg total daily dose) were given together twice daily, 7–8 h apart. PaL and BDQ or TBAJ-876 were given 4 h apart. All single-drug regimens were given for only 1 month while combination regimens were given for 2 months.

### Evaluation of drug efficacy.

Efficacy determinations were based on lung CFU counts after 1 and 2 months of treatment. At each time point, lungs were removed aseptically and homogenized in 2.5 ml PBS. Lung homogenates were plated in serial dilutions on 0.4% charcoal-supplemented 7H11 agar with selective antibiotics: cycloheximide (20 μg/ml), carbenicillin (100 μg/ml), polymyxin B (400,000 U/ml), and trimethoprim (40 μg/ml).

### Evaluation of resistance selection.

At D0, M1, and M2, lung homogenates from groups receiving combination therapy with PaL, BPaL, or SPaL were plated in parallel on Pa- and B-containing plates to quantify the drug-resistant subpopulations. At D0, one aliquot of the lung homogenates from H37Rv-infected mice was plated undiluted and at a 100-fold dilution on one charcoal-free 7H11 agar plate containing Pa 2 μg/ml and one charcoal-free plate containing B 0.125 μg/ml. The same aliquots of the lung homogenates from mutant-infected mice were plated on one charcoal-free plate containing Pa 2 μg/ml and one charcoal-free plate containing B 1 μg/ml to assess *atpE*-mediated resistance (37). At months 1 and 2, one aliquot of the lung homogenates from H37Rv-infected mice was plated undiluted on one charcoal-free plate containing B 0.06 μg/ml. Although this concentration is below the recommended critical concentration of 0.25 μg/ml for clinical use, it enables more effective recovery of *Rv0678* and *pepQ* mutants with small shifts in drug susceptibility (i.e., 2–8× increases in MIC) from lung homogenates of mice, especially when mice are on treatment with BDQ, which accumulates in lungs and is carried over onto plates, increasing the effective concentration of BDQ, as previously described (37, 47). Based on this prior knowledge of appropriate BDQ concentrations for quantification of *Rv0678* and *pepQ* mutants in mouse lung homogenates and because BDQ and TBAJ-876 cross-resistance is conferred by the same mutations at the shared binding site on the c subunit of the F-ATP synthase (43), diarylquinoline-resistant mutants were quantified using BDQ-containing agar and not TBAJ-876-containing agar. Pa resistance was unexpected and difficult to assess during combination therapy against the wild-type H37Rv strain due to BDQ carryover and hence was not assessed in H37Rv-infected mice.

### Whole genome sequencing.

Samples of genomic DNA were sequenced at the Johns Hopkins University Sydney Kimmel Comprehensive Cancer Center Experimental and Computational Genomics Core using an Illumina NovaSeq6000 S4 sequencer (150 bp PE). DNA samples were prepared for sequencing using the TruSeq DNA Kit. Sequencing was performed with 200× coverage. Illumina's CASAVA 1.8.4 was used to convert BCL files to FASTQ files. Default parameters were used. Analysis was done using PATRIC web resources (48). Briefly, BWA-MEM was used for running the alignments. The default parameters were used. The data were aligned to the genome of H37Rv as a reference sequence, NCBI accession number NC_000962-3. Freebayes was used to generate high quality variants for the read library.

### Statistical analysis.

Group mean CFU counts were compared by one-way ANOVA with Bonferroni’s correction to control for multiple comparisons using GraphPad Prism version 6.
