# Supplementary material for: Comparative Efficacy of the Novel Diarylquinoline TBAJ-876 and Bedaquiline against a Resistant Rv0678 Mutant in a Mouse Model of Tuberculosis
Source: Antimicrob Agents Chemother. 2021 Nov 17;65(12):e01412-21. doi: 10.1128/AAC.01412-21 (PMC8597756; doi:10.1128/AAC.01412-21)
Supplement: Supplemental file 1 — Supplemental tables. Download aac.01412-21-s0001.pdf, PDF file, 0.06 MB [file aac.01412-21-s0001.pdf]

Supplementary Data

Table S1: Experimental scheme

Regimen	Time point and No. of mice sacrificed				
	D-13	D0	M1	M2	Total
Untreated	2	3	3		8
B_{25}			5		5
$S_{3.125}$			5		5
$S_{6.25}$			5		5
$S_{12.5}$			5		5
PaL			5	5	10
B_{25}PaL			5	5	10
$S_{3.125}$PaL			5	5	10
$S_{6.25}$PaL			5	5	10
$S_{12.5}$PaL			5	5	10
Total	2	3	48	25	78

[a]Time points shown as days (D) or months (M) of treatment.

Abbreviations: B=bedaquiline; Pa=pretomanid; L=linezolid; S=TBAJ-876. Doses are as follows, Pa = 30 mg/kg, L = 50 mg/kg; other doses are indicated in subscripts after drug abbreviation. Dosing was once daily except for Pa and L which were dosed twice daily.

The same experiment was performed in mice infected with either wild type *M. tuberculosis* H37Rv or a B-resistant *M. tuberculosis* strain with an *Rv0678* mutation.

Table S2: Lung CFU counts in mice infected with wild type *M. tuberculosis* H37Rv

Regimen	Mean (±SD) log_{10} CFU count at[a]:				
	D-13	D0	W3	M1	M2
Untreated	4.07 ± 0.07	7.61 ± 0.22	9.22 ± 0.28[b]		
B$_{25}$				4.46 ± 0.10	
S$_{3.125}$				4.37 ± 0.48	
S$_{6.25}$				3.18 ± 0.21	
S$_{12.5}$				2.36 ± 0.17	
PaL				6.42 ± 0.14	3.73 ± 0.42
B$_{25}$PaL				3.96 ± 0.33	0.41 ± 0.28
S$_{3.125}$PaL				4.14 ± 0.14	0.12 ± 0.16
S$_{6.25}$PaL				2.66 ± 0.37	0.10 ± 0.21
S$_{12.5}$PaL				1.62 ± 0.32	0

[a]Time points shown as days (D), weeks (W), or months (M) of treatment.
[b]Untreated mice were euthanized at Week 3 of the treatment period (5 weeks after infection)
Abbreviations: B=bedaquiline; Pa=pretomanid; L=linezolid; S=TBAJ-876. Doses are as follows, Pa = 30 mg/kg, L = 50 mg/kg; other doses are indicated in subscripts after drug abbreviation. Dosing was once daily except for Pa and L which were dosed twice daily.

Table S3: Lung CFU counts in mice infected with *M tuberculosis Rv0678* mutant (IS6110 insertion at aa16)

Regimen	Mean (±SD) log_{10} CFU count at[a]:				
	D-13	D0	W3	M1	M2
Untreated	4.05 ± 0.01	7.17 ± 0.09	8.84 ± 0.15[b]		
B_{25}				7.19 ± 0.12	
$S_{3.125}$				6.69 ± 0.11	
$S_{6.25}$				5.79 ± 0.23	
$S_{12.5}$				5.05 ± 0.15	
$Pa_{30}L_{50}$				6.64 ± 0.15	4.40 ± 0.35
B_{25}PaL				5.16 ± 0.19	3.47 ± 0.28
$S_{3.125}$PaL				5.06 ± 0.11	2.60 ± 0.27
$S_{6.25}$PaL				4.09 ± 0.26	1.24 ± 0.76
$S_{12.5}$PaL				3.81 ± 0.34	0.83 ± 0.28

[a]Time points shown as days (D), weeks (W), or months (M) of treatment.
[b]Untreated mice were euthanized at Week 3 of the treatment period (5 weeks after infection)

Abbreviations: B=bedaquiline; Pa=pretomanid; L=linezolid; S=TBAJ-876. Doses are as follows, Pa = 30 mg/kg, L = 50 mg/kg; other doses are indicated in subscripts after drug abbreviation. Dosing was once daily except for Pa and L which were dosed twice daily.

Table S4: Proportions of mice and mean log_{10} CFU (±SD) of *M. tuberculosis* CFU showing resistance to bedaquiline (BDQ) 0.06 µg/ml after infection with wild-type *M. tuberculosis* and antimicrobial treatment

	Proportion of mice with detectable BDQ-resistant subpopulations and counts on drug-containing plates (mean frequency of resistant CFU among all CFU recovered)*				
	M1			M2	
Drug, dose (mg/kg)	Monotherapy	DARQ combined with PaL		DARQ combined with PaL	
	BDQ-resistant	BDQ-resistant	PMD-resistant	BDQ-resistant	PMD-resistant
None	NT	4/5<br>($8.4x10^{-3}$)<br>2.31 ± 0.13	NT	3/5<br>($5.4x10^{-3}$)<br>0.92 ± 0.60	NT
BDQ (25)	1/5<br>($1.29x10^{-2}$)<br>2.52	0/5	NT	0/5	NT
TBAJ-876 (3.125)	0/5	0/5	NT	0/5	NT
TBAJ-876 (6.25)	0/5	0/5	NT	0/5	NT
TBAJ-876 (12.5)	0/5	0/5	NT	0/5	NT

Abbreviations: BDQ=bedaquiline; PMD, Pa=pretomanid; L=linezolid; M1 and M2, months of treatment; NT=not tested

At Day 0: Mean (±SD) CFU counts on plates containing no drug, 0.06 µg/ml BDQ and 2 µg/ml PMD were: 7.61±0.22, 2.26±0.30 and 3.07±0.64 log_{10} CFU, respectively.

*reported frequency and CFU counts include only mice in which resistant subpopulations were detected

Table S5: Proportions of mice and mean $\log_{10}$ CFU (±SD) of *M. tuberculosis* CFU showing resistance to bedaquiline (BDQ, 1 µg/ml) and pretomanid (PMD, 2 µg/ml) after infection with the *M. tuberculosis Rv0678* mutant and antimicrobial treatment

	Proportion of mice with detectable BDQ- or PMD-resistant subpopulations and mean (±SD) CFU counts on drug-containing plates (mean frequency of resistant CFU among all CFU recovered)*					
	M1				M2	
Drug, dose (mg/kg)	Monotherapy		DARQ combined with PaL		DARQ combined with PaL	
	BDQ-resistant	PMD-resistant	BDQ-resistant	PMD-resistant	BDQ-resistant	PMD-resistant
None	NT	NT	2/5<br>(6.4×10^{-6})<br>1.60, 1.30	5/5<br>(2.4×10^{-5})<br>1.54 ± 0.77	0/5	4/5<br>(3.6×10^{-4})<br>0.89 ± 0.24
BDQ (25)	0/5	4/5<br>(4.4×10^{-5})<br>2.97±2.0	0/4	1/4<br>(9.1×10^{-5})<br>1.31	0/5	0/5
TBAJ-876 (3.125)	0/5	1/5<br>(1.4×10^{-6})<br>0.70	0/5	2/5<br>(8.8×10^{-4})<br>0.70, 2.41	0/5	2/5<br>(2.6×10^{-2})<br>0.70, 1.00
TBAJ-876 (6.25)	0/5	0/5	0/5	0/5	0/5	0/5
TBAJ-876 (12.5)	0/5	0/5	0/5	0/5	0/5	0/5

Abbreviations: BDQ=bedaquiline; PMD, Pa=pretomanid; L=linezolid; M1 and M2, months of treatment; NT=not tested

At Day 0: Mean (±SD) CFU counts on plates containing no drug, 1 µg/ml BDQ and 2 µg/ml PMD were: 7.17±0.09, 1.99±0.77 and 2.65±1.04 $\log_{10}$ CFU, respectively.

*reported frequency and CFU counts include only mice in which resistant subpopulations were detected.

When only 1 or 2 mice harbored resistant CFU, individual CFU counts (without ±SD) are shown for each mouse.
